# A cross-species multi-omics analyze uncovers conserved molecular mechanisms underlying age-related erectile dysfunction

**DOI:** 10.1093/sexmed/qfaf078

**Published:** 2025-10-17

**Authors:** Qing Long, Yuanhua Jiang, Jun Zhou, Jingxuan Peng

**Affiliations:** Center of Reproductive Medicine, Nanxishan Hospital of Guangxi Zhuang Autonomous Region, Guilin 541000, China; Center of Reproductive Medicine, Nanxishan Hospital of Guangxi Zhuang Autonomous Region, Guilin 541000, China; Department of Andrology, First Hospital of Hunan University of Chinese Medicine, Changsha, Hunan 410000, China; Department of Urology, First Affiliated Hospital of Jishou University, Jishou, Hunan 416000, China

**Keywords:** aging, erectile dysfunction, cross-species, multi-omics, mitochondrial function, extracellular matrix

## Abstract

**Background:**

The urgent need for new treatments is driven by the challenging clinical situation of age-related erectile dysfunction (ARED).

**Aim:**

To clarify the conserved molecular mechanisms of ARED across species using multi-omics.

**Methods:**

Rat and mouse models with ARED were developed to facilitate the extraction of mRNA and proteins from the corpus cavernosum for high-throughput sequencing. Bioinformatics techniques were employed to analyze differentially expressed genes and to conduct analyses using the Kyoto Encyclopedia of Genes and Genomes, Gene Ontology, and protein–protein interaction networks. Verification of the results was carried out using immunofluorescence, hematoxylin–eosin staining, and Masson staining.

**Outcomes:**

The multi-omics profiles of ARED rats and mice were analyzed and validated across species.

**Results:**

In both species, Kyoto Encyclopedia of Genes and Genomes and Gene Ontology analyses of transcriptomic and proteomic data revealed that differentially expressed genes were predominantly enriched in pathways associated with alterations in extracellular matrix composition, downregulation of mitochondrial activity, and disruption of protein homeostasis. Immunofluorescence analysis demonstrated an upregulation of reactive oxygen species expression, coupled with a downregulation of Aldh18a1, collagen, and collagen I expression in the corpus cavernosum of mice and rats with ARED.

**Clinical Implications:**

To offer a novel approach for enhancing the erectile function in patients with ARED.

**Strengths and Limitations:**

The primary strength of this study lies in its utilization of cross-species multi-omics sequencing, which has elucidated the conserved molecular mechanisms underlying ARED. However, a significant limitation is the absence of subsequent validation in patients with ARED.

**Conclusions:**

Cross-species multi-omics comparisons present a potentially innovative approach for elucidating the underlying mechanisms and identifying preventive and therapeutic targets for ARED.

## Introduction

Erectile dysfunction (ED) is a common male sexual disorder characterized by the persistent inability to achieve or maintain an erection sufficient for satisfactory sexual performance.^1^ The global incidence of ED is increasing annually, significantly affecting men’s quality of life and mental health.^2^ With the acceleration of population aging, age-related erectile dysfunction (ARED) has emerged as a critical public health concern.^3^ ARED specifically pertains to ED in older populations and involves a multifaceted pathogenesis that encompasses vascular, neurological, endocrine, and psychological factors.^4-6^ Despite advancements in research, the precise molecular mechanisms underlying ARED remain inadequately understood, and numerous challenges persist in the identification of effective therapeutic targets.

Current therapeutic approaches for ARED predominantly encompass phosphodiesterase type 5 inhibitors (PDE5is), testosterone replacement therapy, and surgical interventions.^7^^,^^8^ Nevertheless, PDE5is demonstrate ineffectiveness in 30% to 50% of patients and are associated with adverse effects, including headache and facial flushing.^9^ Testosterone replacement therapy has the potential to exacerbate prostate hyperplasia or stimulate the progression of prostate cancer.^10^^,^^11^ Furthermore, most existing treatments primarily alleviate symptoms rather than address the underlying vascular, neurological, or metabolic pathologies.^12^^,^^13^ The advancement of etiology-based precision therapies remains in its nascent stages, highlighting the need to identify new therapeutic targets and improve current treatments.^14^ Such efforts are essential for improving treatment efficacy and enhancing the quality of life for patients with ARED.

Transcriptomics facilitates the elucidation of gene expression profiles specific to particular cells or tissues under defined conditions, thereby illuminating the dynamic alterations in gene activity at the transcriptional level.^15^ This approach enhances our understanding of the regulatory mechanisms governing gene expression during the development of ARED. In parallel, proteomics concentrates on the analysis of protein expression levels, modification states, and interactions within cells or tissues.^16^ This discipline is intrinsically linked to cellular structure and function, proving to be invaluable for elucidating the pathophysiological processes associated with ARED. In the context of animal models, rats and mice are extensively employed in ARED research due to their physiological and genetic similarities to humans, as well as their amenability to experimental manipulation.^14^^,^^17^ Comprehensive studies have demonstrated that aged rodents exhibit ED that parallels the condition observed in humans, with numerous molecular-level pathological mechanisms mirroring those present in human ARED.^14^^,^^17^

In this study, we aim to integrate transcriptomic and proteomic technologies to conduct a comprehensive molecular analysis of experimental disease models in aged rats and mice. By examining various facets, including gene expression and protein synthesis/modification, we seek to elucidate the pathogenesis of ARED. Our objective is to identify differentially expressed genes (DEGs) and proteins, construct molecular regulatory networks, and provide novel theoretical foundations and research directions for the development of precision treatment strategies for ARED.

## Methods

### Animal model

Animal studies were conducted in accordance with the Declaration of Helsinki and the Guide for the Care and Use of Laboratory Animals, receiving approval from the Ethics Committees for Animal Experimentation at the the First Hospital of Hunan University of Chinese Medicine (#202403013). All animals were supplied by GemPharmatech and were bred and acclimated in the Animal Facilities of the First Hospital of Hunan University of Chinese Medicine. To assess for ARED, an apomorphine (APO) test was performed. APO, obtained from Sigma, was prepared in a saline solution containing 0.2 mg/mL ascorbic acid and was administered subcutaneously in the dorsal neck region at dosages of 80 μg/kg for rats and 100 μg/kg for mice. Penile erections were monitored within 30 min post-injection; animals that did not exhibit erections during this timeframe were classified as having ARED. This study utilized male Sprague–Dawley rats aged 3 months (young, *n* = 5) and 20 months (ARED, *n* = 5), as well as male C57BL/6N mice aged 2 months (young, *n* = 5) and 18 months (ARED, *n* = 5).

### Differential expression analysis

The differential expression of genes was analyzed using DESeq2 (v1.38.3) under the following criteria: a |log2FoldChange| > 1.5 and a significant *P*-value of <.05. Concurrently, we employed the ComplexHeatmap (v2.16.0) software package to conduct bidirectional clustering analysis of all DEGs across the samples. A heatmap was generated to illustrate the expression levels of individual genes in various samples, as well as the expression patterns of different genes within the same sample. The Euclidean method was utilized to calculate distances, and the Complete Linkage method was applied for clustering.

### Interaction analysis of differential gene protein network

The STRING database (available at https://string-db.org/) is utilized for the analysis of protein interactions to elucidate the relationships among target genes.

### Enrichment analysis

We systematically annotated all genes with terms from the Gene Ontology (GO) database and quantified the number of differentially enriched genes associated with each term. GO enrichment analysis of DEGs, including all DEGs, upregulated DEGs, and downregulated DEGs, was conducted using the ClusterProfiler software (version 4.6.0). The hypergeometric distribution method was employed to calculate *P*-values, with a threshold for significant enrichment set at *P*-value <.05. This analysis identified GO terms with significantly enriched differential genes, thereby elucidating the primary biological functions associated with these genes. Additionally, ClusterProfiler (version 4.6.0) was utilized to perform enrichment analysis of the Kyoto Encyclopedia of Genes and Genomes (KEGG) pathways for the differential genes, emphasizing pathways with significant enrichment at *P*-value <.05.

### Gene Set EnrichmentAnalysis

The Gene Set EnrichmentAnalysis (GSEA) enrichment analysis of all genes was conducted using the ClusterProfiler tool (version 4.6.0), and a pathway map of the GSEA enrichment analysis was subsequently generated.

### Antibodies

The following primary antibodies were used: Aldh18a1 (68184-1-Ig, Proteintech), collagen I (A1352, ABclonal).

### Reactive oxygen species detection

Reactive oxygen species (ROS) levels were assessed using a commercial ROS detection kit (C10422). Fresh penile tissues were snap-frozen, cryosectioned into 5 μm slices, and stained with CellROX Deep Red (1 μM) together with DAPI for 30 min at 37°C in a light-protected, humid chamber. Images were then captured under a Leica DMi8 fluorescence microscope.

### Immunofluorescence

Following fixation, the penile tissues were sectioned into paraffin slices with a thickness of 5 μm. The tissue sections were then fixed using an appropriate fixative for 10-15 min at room temperature. After permeabilizing the cells to facilitate antibody penetration through the cell membrane, the diluted primary antibody was added to the samples, which were subsequently incubated overnight at 4°C. Visualization of the samples was performed using a Leica DMi8 microscope after treatment with a secondary antibody for 1 h at room temperature in the dark.

### Histological examination of corpus cavernosum

After fixation, the penile tissues were sectioned into paraffin slices with a thickness of 5 μm and subsequently stained using Masson’s trichrome stain.

### Statistical analysis

The Shapiro–Wilk test was used to evaluate whether the data distribution was normal. The Mann–Whitney *U* test was utilized for non-normally distributed data when comparing two groups, whereas the Student’s *t*-test was used for normally distributed data. GraphPad Prism software (version 8) was used for statistical analyses and visualizations, with results presented as mean ± SD.

## Results

### Analysis of homologous genes and DEGs in transcriptomics

**Figure 1:**
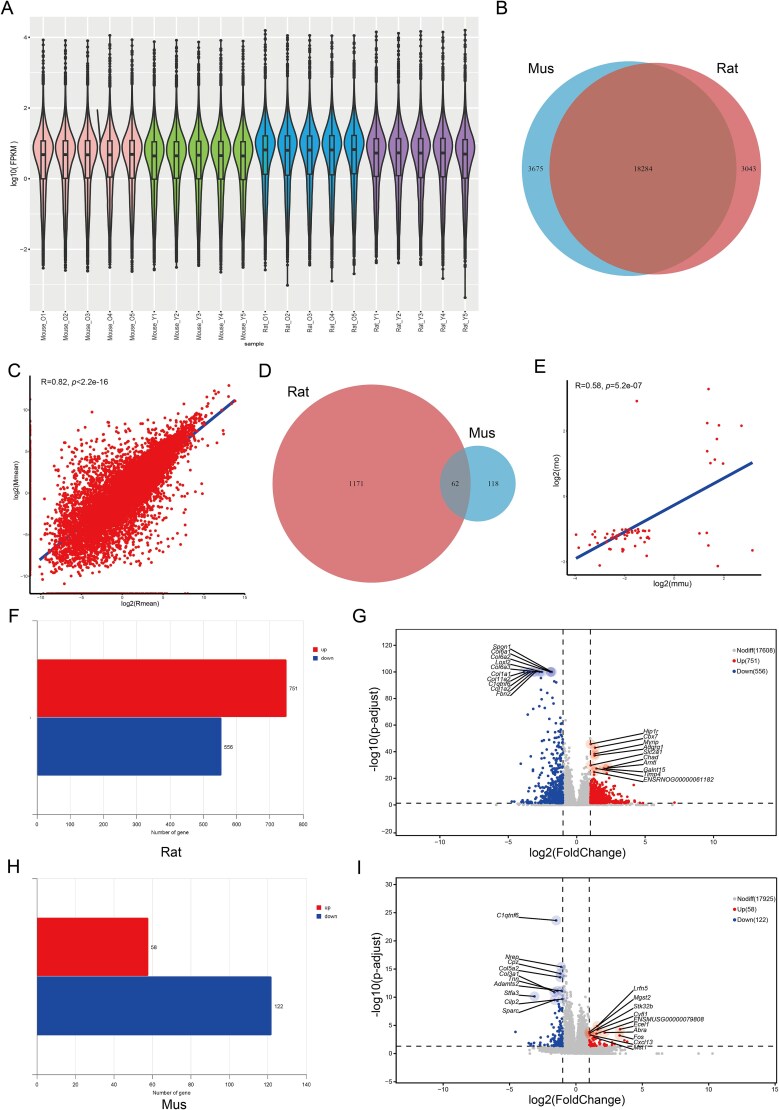
Analysis of homologous genes and DEGs in transcriptomics. (A) Violin plots displaying protein-coding gene expression profiles in the corpus cavernosum of rats and mice. (B) Venn diagram showing the set of homologous genes shared between rat and mouse corpus cavernosum. (C) Pearson correlation analysis evaluating homologous gene expression relationships in the corpus cavernosum of normal rats and mice. (D) Venn diagram representing the intersection of DEGs identified in ARED rats and mice. (E) Scatter plot illustrating the correlation of shared DEGs between the two species. (F and G) Bar chart and volcano plot comparing DEGs in ARED rats with those in the control group. (H and I) Bar chart and volcano plot comparing DEGs in ARED mice with those in the control group.

In the mouse corpus cavernosum, gene expression appeared more evenly distributed than in rats (Figure 1A). To maintain comparability, analysis was restricted to 18 284 orthologous genes (Figure 1B). Correlation assessment revealed a high degree of similarity between the two species, with a Pearson’s *r* of 0.82 and a *P*-value below 2.2 × 10^−16^ (Figure 1C).

A total of 62 homologous DEGs were obtained by intersecting the DEG sets from both species, enabling the investigation of their shared molecular alterations (Figure 1D). These homologous DEGs were classified into four patterns: genes upregulated in both species, genes downregulated in both, genes upregulated in rats but downregulated in mice, and genes downregulated in rats but upregulated in mice. Such distribution reflects both overlapping and species-specific molecular changes during ARED progression (Figure 1E).

RNA-seq data from rats and mice were analyzed using the DESeq2 package, revealing 1233 DEGs in ARED rats. Among them, 751 genes (60.91%) showed upregulation, a proportion slightly exceeding that observed in the control group (Figure 1F and G). In ARED mice, we found 180 DEGs compared to the normal group, with 122 (67.78%) being downregulated and 58 (32.22%) being upregulated (Figure 1H and I).

Given that conserved alterations between species may pinpoint key factors in disease initiation and progression, we proposed that same trend differentially expressed genes (ST-DEGs) could more effectively represent shared biological changes. Therefore, ST-DEGs from both the homologous upregulated and homologous downregulated groups were selected for detailed bioinformatics analysis, aiming to uncover pivotal molecular pathways and potential therapeutic targets for ARED.

### Analysis of functional enrichment for upregulated homologous DEGs in the transcriptome

**Figure 2:**
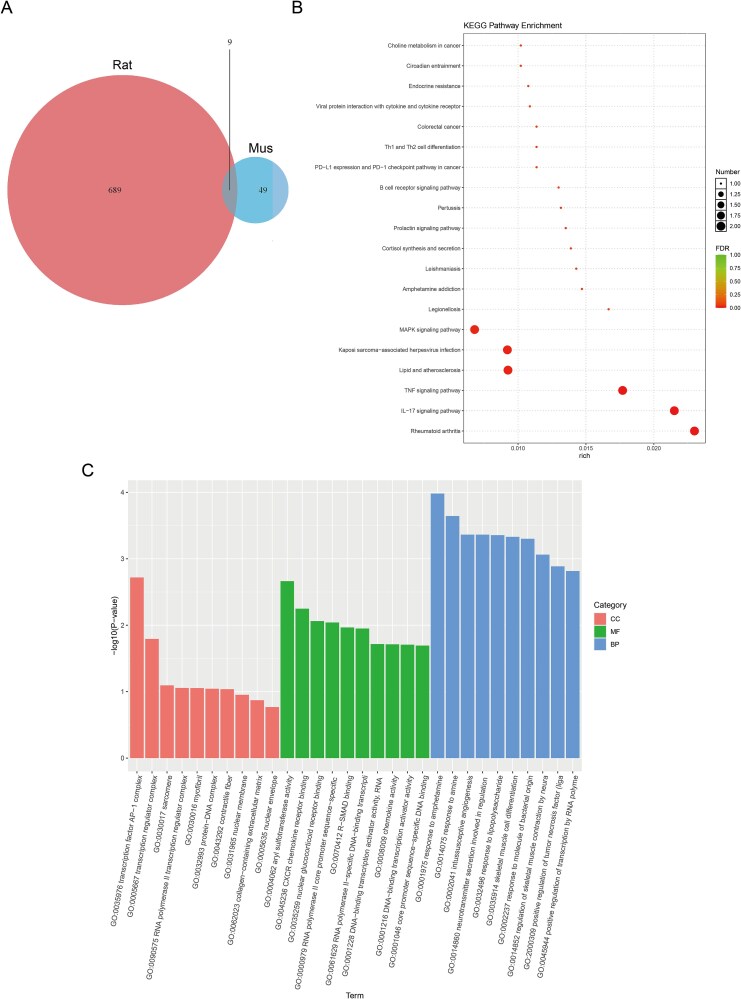
Enrichment analysis of homologous genes with upregulated expression in transcriptomic data. (A) Venn diagram showing DEGs consistently upregulated in both ARED rats and mice. (B) KEGG pathway enrichment results for co-upregulated DEGs. (C) GO enrichment results for co-upregulated DEGs. CC: cellular component; BP: biological process; MF: molecular function.

Within the ARED and control group comparison across both species, nine homologous DEGs exhibiting upregulation were identified (Figure 2A and Supplementary Figure S1A). Supplementary Figure S1A presents a heatmap visualization of DEGs associated with rheumatoid arthritis that are conserved across species. This visualization further substantiates the involvement of pro-inflammatory pathways, including interleukin-17 (IL-17) and tumor necrosis factor (TNF) signaling, in ARED. KEGG pathway analysis revealed significant enrichment of these genes in rheumatoid arthritis–related pathways, as well as in the IL-17 and TNF signaling cascades (Figure 2B). GO enrichment analysis revealed that these genes were mainly associated with biological processes like response to amphetamine and amine (Figure 2C).

### Analysis of functional enrichment for homologous DEGs with decreased expression in the transcriptome

**Figure 3:**
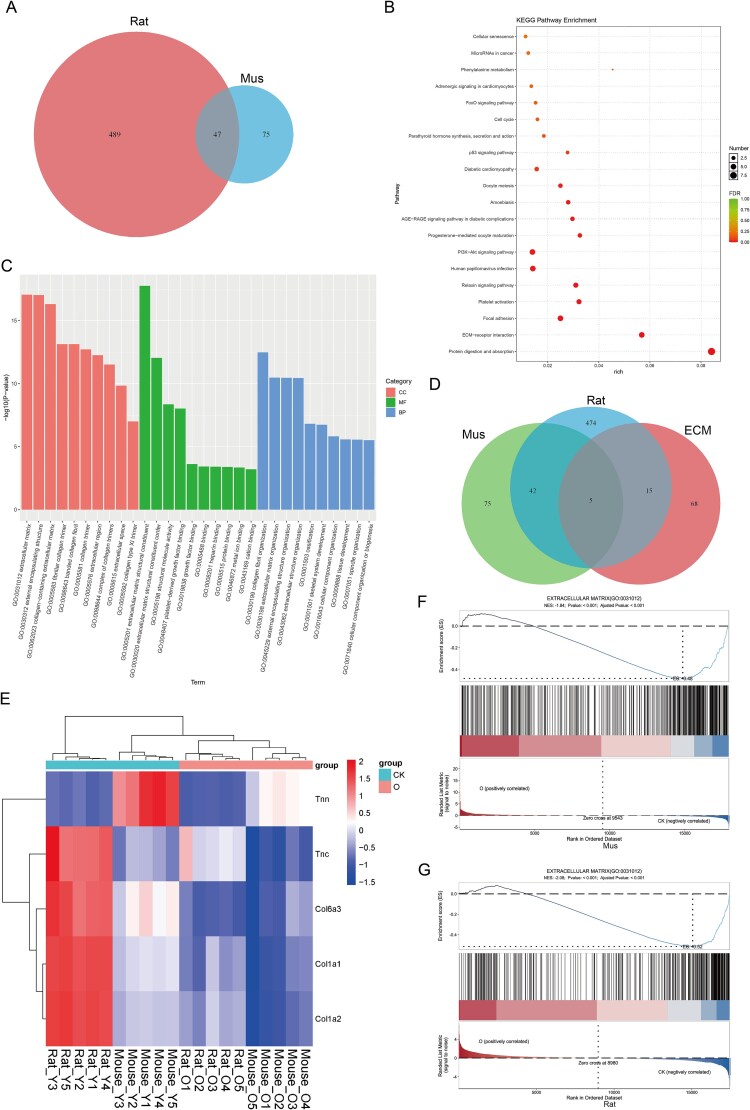
Enrichment analysis of downregulated homologous genes in transcriptomics. (A) The Venn diagram illustrates the DEGs that are downregulated in both ARED rats and mice. (B) KEGG analysis of co-downregulated DEGs. (C) GO enrichment analysis of co-downregulated DEGs. (D) Venn diagram illustrating DEGs shared by rats, mice, and ECM-related genes. (E) Heatmap displaying hierarchical clustering of shared ECM-related DEGs in rats and mice. (F) GSEA analysis of ECM pathway in mice. (G) GSEA analysis of ECM pathway in rats. CC: cellular component; BP: biological process; MF: molecular function.

In a similar manner, we found 47 co-regulated DEGs among the 536 DEGs that were upregulated in rats and the 122 DEGs that were downregulated in mice (Figure 3A). KEGG enrichment analysis of these 47 DEGs indicated that they were mainly enriched in pathways associated with protein digestion and absorption, the extracellular matrix (ECM), and focal adhesion (Figure 3B). GO enrichment analysis also suggested that the downregulated genes related to CC were mainly linked to ECM, external encapsulating structure, and collagen-containing ECM. Their molecular functions (MFs) were linked to the structural components of the ECM, with biological processes connected to collagen fibril organization, ECM organization, and the organization of extracellular encapsulating structures (Figure 3C). The analysis uncovered five ECM-related DEGs showing a downregulation trend (Figure 3D and E). The GSEA plots for both rats and mice (Figure 3F and G) revealed a significant negative enrichment of the ECM pathway, thereby further substantiating its global downregulation in ARED models. These results align with our KEGG and GO enrichment analyses, reinforcing the concept that ECM remodeling is a conserved pathological characteristic in ARED.

### GO and KEGG enrichment analysis of upregulated DEPs from proteomic

**Figure 4:**
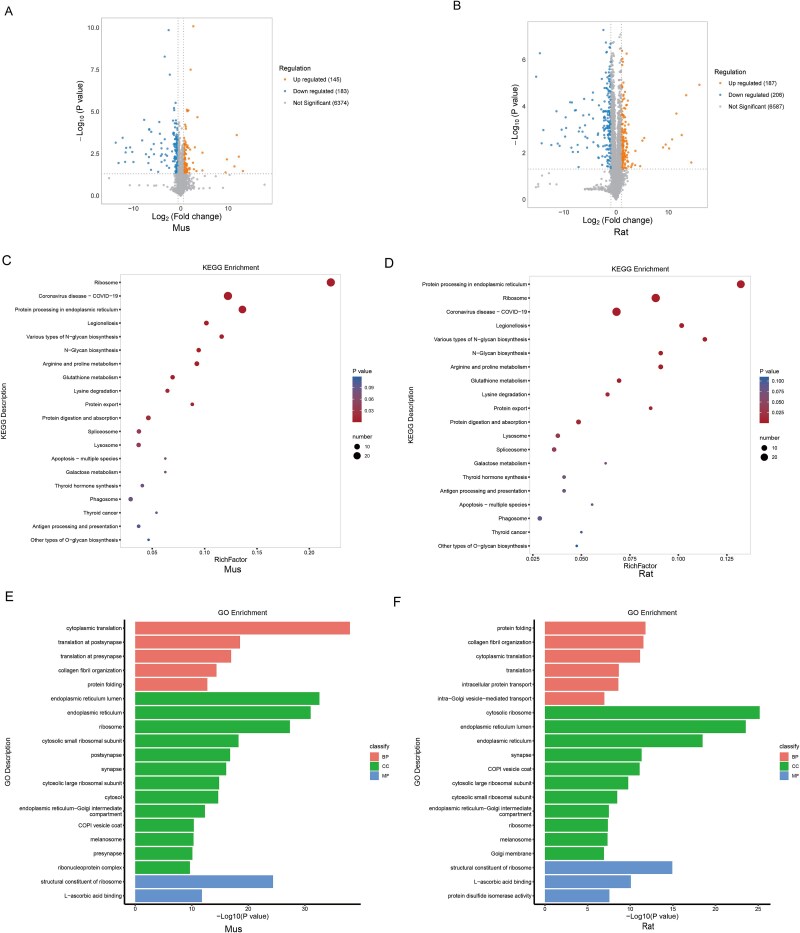
Enrichment analysis of upregulated genes in proteomics. (A and B) The volcano plots show the upregulated DEGs in ARED rats and mice. (C and D) KEGG analysis of upregulated DEGs in ARED rats and mice. (E and F) GO enrichment analysis of upregulated DEGs in ARED rats and mice. CC: cellular component; BP: biological process; MF: molecular function.

Proteomic analysis revealed 328 regulated DEGs, consisting of 145 upregulated and 183 downregulated DEGs in mice (Figure 4A). In rats, there were 187 DEGs that were upregulated, along with 206 other downregulated DEGs (Figure 4B). For the upregulated DEGs, KEGG enrichment analysis in both species revealed significant enrichment in pathways associated with the protein processing in the endoplasmic reticulum and ribosome (Figure 4C and D). In both species, GO enrichment analysis revealed that the upregulated genes related to biological process (BP) were linked to cytoplasmic translation, collagen fibril organization, and protein folding. Their CC was mainly linked to the endoplasmic reticulum lumen, endoplasmic reticulum, ribosome, small and large cytosolic ribosomal subunits, and synapse, with MF involving ribosome structure and l-ascorbic acid binding (Figure 4E and F).

### GO and KEGG enrichment analysis of downregulated DEPs from proteomic

**Figure 5:**
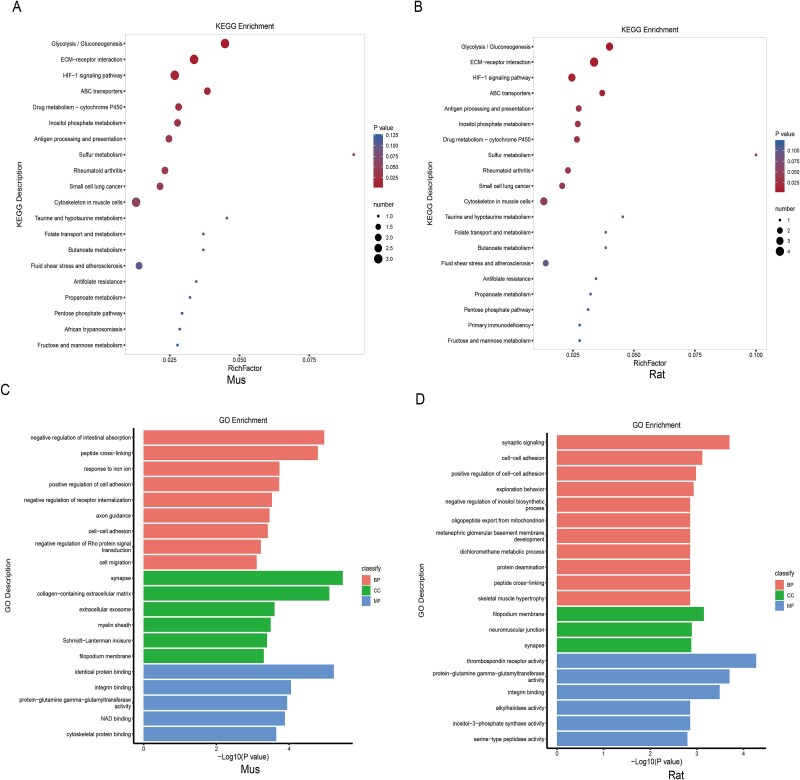
Enrichment analysis of downregulated genes in proteomics. (A and B) KEGG analysis of downregulated DEGs in ARED rats and mice. (C and D) GO enrichment analysis of downregulated DEGs in ARED rats and mice. CC: cellular component; BP: biological process; MF: molecular function.

The KEGG enrichment analysis revealed that both species showed significant downregulation of DEPs in pathways related to glycolysis/gluconeogenesis, ECM–receptor interaction, and the HIF-1 signaling pathway (Figure 5A and B). For both species, GO enrichment analysis identified that the downregulated genes concerning BP were tied to peptide cross-linking and cell–cell adhesion. Their CC was mainly linked to synapse with MF concerning recognition, integrin binding, and protein-glutamine gamma-glutamyltransferase activity (Figure 5C and D).

### Detection and verification of DEGs linked to mitochondria

**Figure 6:**
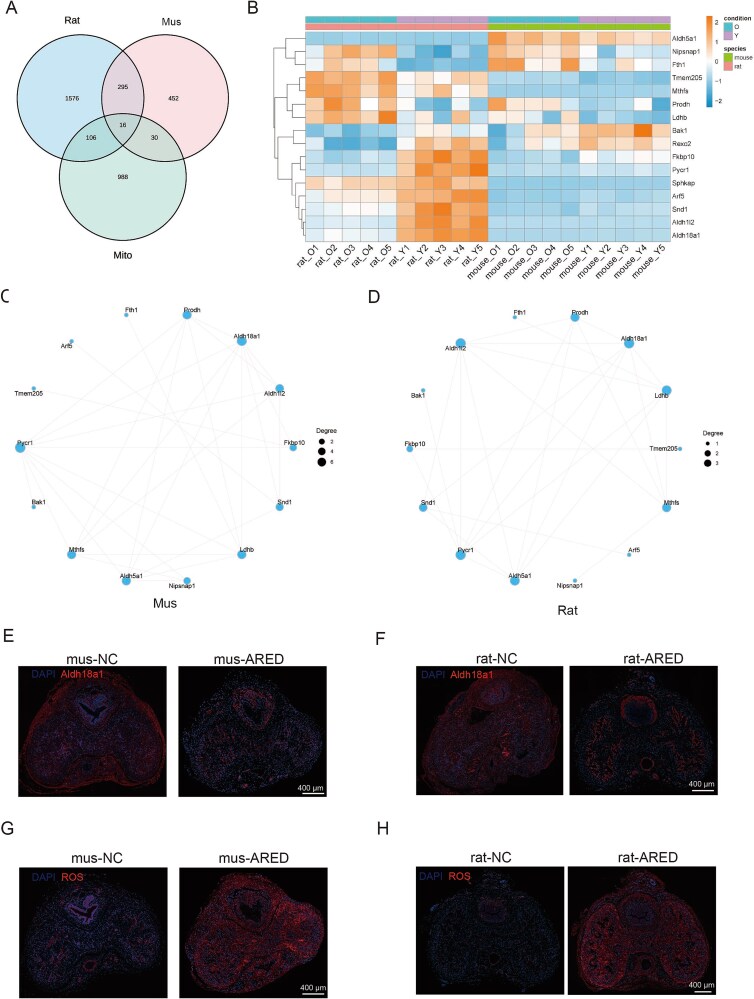
Identification and validation of mitochondria-related DEGs in proteomics. (A) The Venn diagram illustrates the DEGs among rats, mice, and mitochondrial genes. (B) The heatmap illustrates the hierarchical clustering of common mitochondria-related DEGs in rats and mice. Blue denotes low expression levels, while red signifies high expression levels. (C and D) analysis of interaction networks for mitochondria-related DEGs in ARED rats and mice. (E and F) Immunohistochemical analysis was conducted to examine the expression of Aldh18a1 in the corpus cavernosum of both murine and rat models (scale bar = 400 μm). (G and H) The ROS levels in the corpus cavernosum of both mice and rats (scale bar = 400 μm).

Given that proteomic analyses suggest a significant role for mitochondria in ARED, we further investigate the mitochondrial modifications associated with this condition. We extracted a total of 1140 mitochondrial genes from the MitoCarta database^18^ and conducted a comparative analysis with DEGs identified in rat and mouse models. This analysis identified 16 mitochondria-related DEGs (Figure 6A), of which 15 were classified as ST-DEGs. Notably, nine of these ST-DEGs exhibited a trend of downregulation, including *Bak1*, *Arf5*, *Fkbp10*, *Sphkap*, *Snd1*, *Rexo2*, *Aldh1l2*, *Aldh18a1*, and *Pycr1* (Figure 6B). Utilizing these 14 mitochondrial ST-DEGs, we constructed a protein–protein interaction (PPI) network for both rats and mice (Figure 6C and D).

The GSEA further revealed a consistent downregulation of the oxidative phosphorylation pathway in both rat and mouse models (see Supplementary Figure S1B and C). This finding underscores the concept of mitochondrial dysfunction as a conserved pathological hallmark in ARED. Our experimental findings indicate that the expression of *Aldh18a1* was significantly reduced in the ARED model group (Figure 6E and F). Mitochondria serve as the primary source of intracellular ROS, and their dysfunction can lead to abnormalities in the electron transport chain, subsequently resulting in increased ROS production, which is consistent with our observations in ARED samples (Figure 6G and H).

### Detection and verification of DEGs linked to ECM

**Figure 7:**
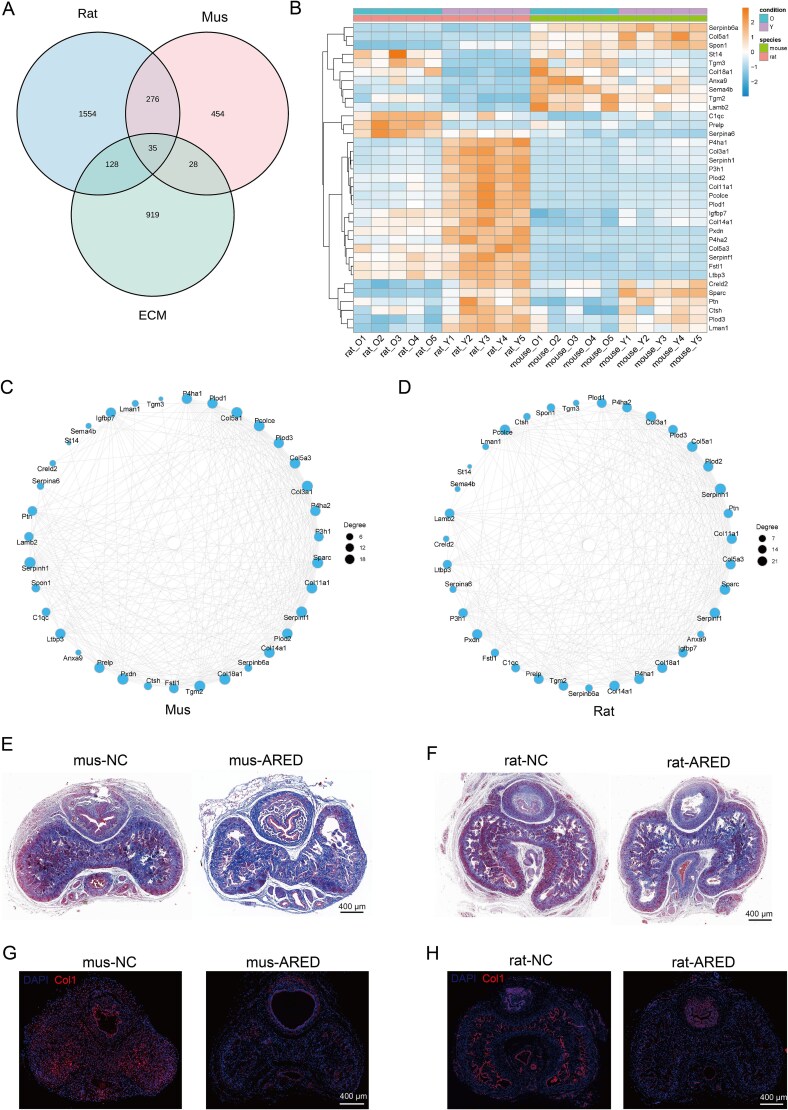
Identification and validation of ECM-related DEGs in proteomics. (A) The Venn diagram illustrates the DEGs among rats, mice, and mitochondrial genes. (B) The heatmap illustrates the hierarchical clustering of common ECM-related DEGs in rats and mice. Blue denotes low expression levels, while red signifies high expression levels. (C and D) Analysis of interaction networks for ECM-related DEGs in ARED rats and mice. (E and F) The Masson staining in the corpus cavernosum of the mice and rats (scale bar = 400 μm). (G and H) Immunohistochemical analysis was conducted to examine the expression of collagen type I in the corpus cavernosum of both murine and rat models (scale bar = 400 μm).

We hypothesized that ECM composition changes during ARED progression based on transcriptomic and proteomic analyses. To explore this, we analyzed 1110 ECM-related genes from Petrov et al.’s study,^19^ identifying 35 ECM-associated DEGs in rats and mice (Figure 7A), with 33 showing the same trend. Most of these (24/33) were downregulated (Figure 7B). Using the STRING database, we constructed a PPI network (Figure 7C and D), revealing that all hub genes were linked to ECM collagen composition, suggesting potential collagen alterations in ARED models. To evaluate this hypothesis, we utilized Masson staining and immunohistochemistry to analyze the ECM components of the corpus cavernosum in animal models of ARED rats and mice. The Masson staining results demonstrated a marked reduction in collagen content within the corpus cavernosum of the ARED groups (Figure 7E and F). Furthermore, immunofluorescence (IF) analysis indicated a downregulation of collagen type I expression in both ARED rats and mice (Figure 7G and H).

## Discussion

As the global population continues to age and the prevalence of age-related diseases increases, the focus of medical research is transitioning from disease treatment to interventions targeting the aging process itself.^20^^,^^21^ Previous research has identified 12 hallmarks of aging: genomic instability, telomere attrition, epigenetic alterations, loss of proteostasis, disabled autophagy, deregulated nutrient sensing, mitochondrial dysfunction, cellular senescence, stem cell exhaustion, altered intercellular communication, chronic inflammation, and dysbiosis.^20^ A recent study has expanded this list by adding two additional characteristics: changes in the ECM and socio-psychological isolation.^21^ Age-related diseases emerge from complex interactions. Cross-species multi-omics analyses not only enhance our understanding of the evolution of biological systems and gene function but also aid in elucidating potential molecular mechanisms underlying diseases.^22^^,^^23^ Despite the availability of several treatment modalities for ARED, such as PDE5is and hormone therapies, their overall efficacy remains constrained. For instance, PDE5is fail to address the underlying vascular or metabolic dysfunctions and are ineffective in a significant proportion of patients. Furthermore, testosterone replacement therapy carries potential risks, including prostate hyperplasia. These limitations highlight the imperative for the development of novel therapeutic strategies that target the fundamental causes of ED in the aging population.^7-13^ In our integrated transcriptomic and proteomic study of ARED in rats and mice, we observed changes in gene and protein expression levels, revealing key molecular features and regulatory mechanisms. These findings highlighted alterations in ECM composition, downregulation of mitochondrial activity, and disruptions in protein homeostasis.

The ECM, a complex assembly of structural and functional molecules secreted by cells, constitutes a major component of the extracellular environment.^24^ An increase in collagen and ECM content has been observed in the penile corpus cavernosum of patients with vasculogenic ED and in diabetic rat models.^15^^,^^25^^,^^26^ Notably, this study identified reduced ECM viscoelasticity as a pathological feature of the corpus cavernosum in ARED. Previous research has demonstrated that alterations in the ECM contribute to the regulation of aging by affecting mitochondrial homeostasis, stem cell activity, and fibrosis.^20^^,^^21^^,^^27-29^ The decline in ECM viscoelasticity is associated with overall aging and influences these processes.^27-30^ In this study, multi-omics, cross-species, and in vivo experiments revealed a reduction in ECM content and mitochondrial activity in ARED. However, the underlying mechanisms in ARED remain unclear. Therefore, elucidating the molecular mechanisms that regulate ECM interactions may be crucial for the development of therapies for ARED. Our research elucidates that mitochondrial dysfunction and ECM remodeling constitute two conserved and interrelated pathological hallmarks of ARED.^20^^,^^27^ Traditionally regarded as distinct processes, emerging evidence—including our findings—underscores a functional interplay between these phenomena. Specifically, mitochondrial impairment results in the excessive production of ROS, which subsequently activate matrix metalloproteinases (MMPs), pivotal enzymes involved in ECM degradation, including the breakdown of collagen I. This ROS–MMP–ECM cascade, extensively implicated in fibrotic diseases, may similarly contribute to the structural deterioration of the corpus cavernosum observed in ARED.^31^^,^^32^

At the core of this regulatory axis is Aldh18a1, a mitochondrial enzyme with a dual function in maintaining redox homeostasis and facilitating proline biosynthesis.^33-35^ Given that proline is a critical precursor for collagen synthesis, the observed downregulation of Aldh18a1 in both rats and mice with ARED may undermine ECM integrity through two primary mechanisms: (1) the exacerbation of oxidative stress, which enhances MMP-mediated matrix degradation, and (2) the impairment of collagen biosynthesis due to proline deficiency. This indicates the existence of an Aldh18a1–ROS–MMP–ECM regulatory loop, whereby mitochondrial dysfunction contributes to both the enzymatic degradation and the biosynthetic insufficiency of the ECM. In addition to Aldh18a1, our study identified several other mitochondrial DEGs that may have significant implications for the pathophysiology of ARED. Notably, the upregulation of Bak1, a pro-apoptotic member of the Bcl-2 family, suggests an increase in mitochondrial-mediated apoptosis within the smooth muscle cells of the corpus cavernosum, potentially exacerbating tissue atrophy and functional decline.^36^^,^^37^ Furthermore, the significant downregulation of Fkbp10 and Pycr1, which are involved in collagen cross-linking and proline metabolism, respectively, supports a model of mitochondrial-driven ECM dysregulation.^38^^,^^39^ Collectively, these findings suggest a mitochondria–collagen axis, wherein compromised mitochondrial function disrupts both cellular survival and matrix homeostasis, thereby accelerating the progression of ARED. Future research should aim to validate this proposed axis through functional in vivo experiments, such as Aldh18a1 overexpression or MMP inhibition, and co-expression network analyses, with the ultimate objective of identifying novel therapeutic targets for ARED treatment.

Current research in ARED has shifted from a singular organ perspective to a multi-system approach that examines the complex interplay of various aging mechanisms.^40^ Clinically, this paradigm is advancing toward precision medicine and personalized treatment strategies, elucidating the interactions among vascular, neurological, inflammatory, and epigenetic factors.^5^^,^^41^^,^^42^ Central to the pathogenesis of ARED are the decline in aging-related endothelial nitric oxide synthase activity—attributable to oxidative stress-induced dephosphorylation—exhaustion of endothelial progenitor cells, and abnormal vascular remodeling resulting from imbalances between matrix MMPs and tissue inhibitors of metalloproteinases, all of which contribute to endothelial dysfunction.^43-47^ Additionally, diminished cholinergic function in the penis, characterized by reduced choline acetyltransferase activity, along with decreased nerve growth factor signaling, exacerbates nerve repair processes.^48-50^ Chronic inflammation, marked by elevated levels of IL-6 and TNF-α, coupled with oxidative stress due to excessive ROS and senescence-associated secretory phenotype in smooth muscle cells, collectively promote fibrosis and dysfunction, thereby perpetuating a vicious cycle of inflammation, oxidation, and aging.^50^^,^^51^ With the integration of aging biology and advanced technologies, there is potential for improved early prediction of ARED, effective interventions, and enhanced long-term maintenance of sexual function in older men. And based on our findings, several potential therapeutic strategies can be proposed. Firstly, the observed downregulation of mitochondrial genes, such as Aldh18a1, alongside elevated ROS levels, suggests that targeting oxidative stress could be a viable intervention. Antioxidant therapies, including Nrf2 activators or mitochondrial-targeted antioxidants like MitoQ, may mitigate ROS-induced damage in the corpus cavernosum. Secondly, the disruption of ECM organization, particularly the reduction in collagen content, underscores ECM as another promising therapeutic target. Agents that enhance ECM synthesis or inhibit its degradation, such as transforming growth factor-β (TGF-β) pathway modulators or collagen-stabilizing peptides, may aid in restoring penile tissue integrity. Furthermore, emerging regenerative approaches, including exosome-derived microRNAs or stem cell therapies, may exert protective effects by modulating both ROS and ECM pathways. These intervention strategies warrant further experimental validation in preclinical models of ARED.

Current research challenges related to ARED encompass several key areas: the limited clinical translation, characterized by a paucity of long-term data on gene and cell therapies; the intricate management of comorbidities, where the interaction mechanisms between diabetes and cardiovascular diseases remain inadequately understood; and an incomplete mechanistic framework, particularly in emerging fields such as the gut microbiota-metabolism axis, which require further exploration.^3^^,^^7^^,^^14^^,^^21^^,^^31^^,^^32^ Future research endeavors should prioritize the development of multi-omics-based risk prediction models, the translation of senescence-targeted therapies (such as SIRT1 activators and NLRP3 inhibitors) and the establishment of a comprehensive management system that integrates prevention, treatment, and rehabilitation.

This study is subject to certain limitations. Firstly, although we have investigated the common molecular alterations in the corpus cavernosum of ARED (androgen receptor deficiency) rats and mice using multi-omics approaches, it remains to be determined whether these processes are conserved in human ARED tissues. Secondly, we concentrated on identifying conserved molecular alterations across species through integrated transcriptomic and proteomic analyses, with subsequent validation via IF and histological staining. Nonetheless, we recognize the limitation posed by the absence of direct functional assays to confirm the causal roles of key targets such as Aldh18a1 and collagen-related genes. In future research, we intend to conduct in vivo knockdown or overexpression experiments utilizing viral vectors or siRNA delivery in aged animal models to ascertain the functional significance of these targets in ARED. These methodologies will facilitate the assessment of whether modulation of these genes can restore mitochondrial function or ECM integrity and enhance erectile function, thereby reinforcing the therapeutic potential of our findings.

In addition to validating animal models, future research should utilize publicly accessible human datasets to evaluate the clinical significance of the conserved DEGs and pathways identified in our study. Large-scale transcriptomic datasets, such as those available from the Gene Expression Omnibus or The Cancer Genome Atlas, could be analyzed to detect expression changes in genes related to the ECM and mitochondria within penile or vascular tissues of aging individuals or patients with ED. Incorporating these human data will facilitate the validation of the conserved molecular mechanisms identified in our cross-species models and assess their translational potential. Moreover, although currently limited, single-cell RNA sequencing datasets of penile tissues may provide valuable insights into cell type-specific alterations and aid in the refinement of therapeutic targets.

## Conclusion

Overall, by integrating transcriptomic and proteomic methodologies, this study systematically delineates the molecular landscape of ARED, accurately identifying key molecules, signaling pathways, and regulatory networks. This lays the groundwork for understanding ARED and identifying targeted treatments, thereby significantly advancing the field of precision medicine for ARED.

## Supplementary Material

Supplement_figure_1_qfaf078

Supplement_Figure_1_caption_qfaf078

## Data Availability

The data used to support the findings of the present study are available from the corresponding author upon request.
