# Supplementary figures and images for: A cross-species multi-omics analyze uncovers conserved molecular mechanisms underlying age-related erectile dysfunction
Source: Sex Med. 2025 Oct 17;13(5):qfaf078. doi: 10.1093/sexmed/qfaf078 (PMC12531994; doi:10.1093/sexmed/qfaf078)

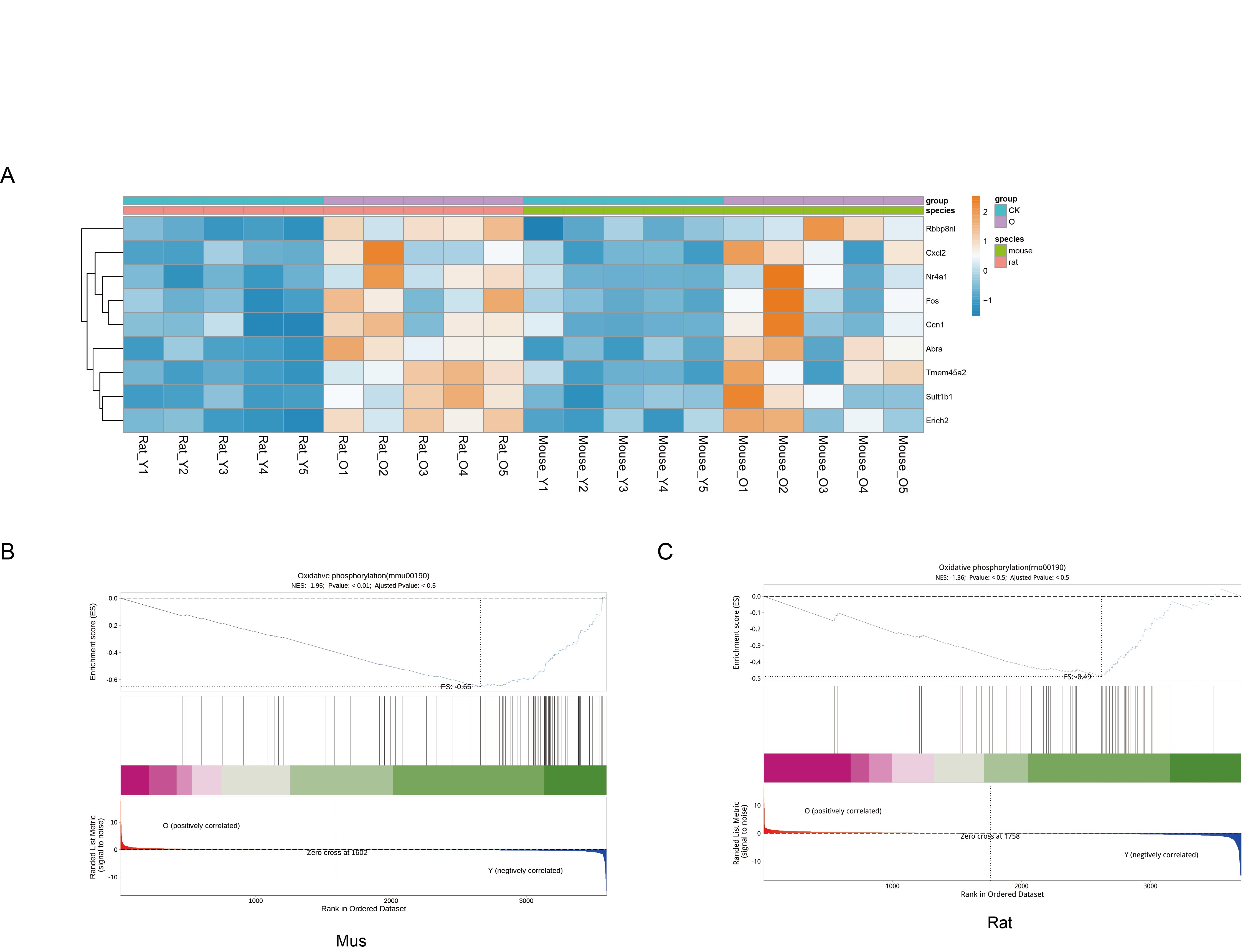

Supplement: Supplement_figure_1_qfaf078 [file supplement_figure_1_qfaf078.jpeg]
