# Supplementary material for: A cross-species multi-omics analyze uncovers conserved molecular mechanisms underlying age-related erectile dysfunction
Source: Sex Med. 2025 Oct 17;13(5):qfaf078. doi: 10.1093/sexmed/qfaf078 (PMC12531994; doi:10.1093/sexmed/qfaf078)
Supplement: Supplement_Figure_1_caption_qfaf078 [file supplement_figure_1_caption_qfaf078.docx]

**Supplement Figure 1**

(A) The heatmap illustrates the hierarchical clustering of common rheumatoid arthritis related DEGs in rats and mice. Blue denotes low expression levels, while red signifies high expression levels.

(B, C) GSEA analysis of oxidative phosphorylation pathway in mice and rats.
